# Caffeic Acid Phenethyl Ester and Ethanol Extract of Propolis Induce the Complementary Cytotoxic Effect on Triple-Negative Breast Cancer Cell Lines 

**DOI:** 10.3390/molecules20059242

**Published:** 2015-05-20

**Authors:** Anna Rzepecka-Stojko, Agata Kabała-Dzik, Aleksandra Moździerz, Robert Kubina, Robert D. Wojtyczka, Rafał Stojko, Arkadiusz Dziedzic, Żaneta Jastrzębska-Stojko, Magdalena Jurzak, Ewa Buszman, Jerzy Stojko

**Affiliations:** 1 Department of Pharmaceutical Chemistry, School of Pharmacy with the Division of Laboratory Medicine in Sosnowiec, Medical University of Silesia in Katowice, Jagiellońska 4, 41-200 Sosnowiec, Poland; E-Mails: annastojko@sum.edu.pl (A.R.-S.); ebuszman@sum.edu.pl (E.B.); 2 Department of Pathology, School of Pharmacy with the Division of Laboratory Medicine in Sosnowiec, Medical University of Silesia in Katowice, Ostrogórska 30, 41-200 Sosnowiec, Poland; E-Mails: adzik@sum.edu.pl (A.K.-D.); rkubina@sum.edu.pl (R.K.); 3 Department of Hygiene, Bioanalysis and Environmental Studies, School of Pharmacy with the Division of Laboratory Medicine in Sosnowiec, Medical University of Silesia in Katowice, Ostrogórska 30, 41-200 Sosnowiec, Poland; E-Mail: amozdzierz@sum.edu.pl; 4 Department and Institute of Microbiology and Virology, School of Pharmacy with the Division of Laboratory Medicine in Sosnowiec, Medical University of Silesia in Katowice, Jagiellońska 4, 41-200 Sosnowiec, Poland; E-Mail: rwojtyczka@sum.edu.pl; 5 Department of Women Health, School of Health Sciences, Medical University of Silesia in Katowice, Medyków 12, 40-752 Katowice, Poland; E-Mail: rstojko@sum.edu.pl; 6 Department of Conservative Dentistry with Endodontics, School of Medicine with the Division of Dentistry in Zabrze, Medical University of Silesia in Katowice, Pl. Akademicki 17, 41-902 Bytom, Poland; E-Mail: adziedzic@sum.edu.pl; 7 Department of Anesthesiology and Intensive Care, School of Medicine, Medical University of Silesia in Katowice, Medyków 14, 40-752 Katowice, Poland; E-Mail: zak@czkstojko.pl; 8 Department of Cosmetology, School of Pharmacy with the Division of Laboratory Medicine in Sosnowiec, Medical University of Silesia in Katowice, Kasztanowa 3, 41-200 Sosnowiec, Poland; E-Mail: magda@sum.edu.pl

**Keywords:** caffeic acid phenethyl ester, propolis, cytotoxicity, breast cancer

## Abstract

Chemotherapy of breast cancer could be improved by bioactive natural substances, which may potentially sensitize the carcinoma cells’ susceptibility to drugs. Numerous phytochemicals, including propolis, have been reported to interfere with the viability of carcinoma cells. We evaluated the *in vitro* cytotoxic activity of ethanol extract of propolis (EEP) and its derivative caffeic acid phenethyl ester (CAPE) towards two triple-negative breast cancer (TNBC) cell lines, MDA-MB-231 and Hs578T, by implementation of the MTT and lactate dehydrogenase (LDH) assays. The morphological changes of breast carcinoma cells were observed following exposure to EEP and CAPE. The IC_50_ of EEP was 48.35 µg∙mL^−1^ for MDA-MB-23 cells and 33.68 µg∙mL^−1^ for Hs578T cells, whereas the CAPE IC_50_ was 14.08 µM and 8.01 µM for the MDA-MB-231 and Hs578T cell line, respectively. Here, we report that propolis and CAPE inhibited the growth of the MDA-MB-231 and Hs578T lines in a dose-dependent and exposure time-dependent manner. EEP showed less cytotoxic activity against both types of TNBC cells. EEP and, particularly, CAPE may markedly affect the viability of breast cancer cells, suggesting the potential role of bioactive compounds in chemoprevention/chemotherapy by potentiating the action of standard anti-cancer drugs.

## 1. Introduction

Breast cancers in women constitute the second most frequent group of cancers worldwide, and their number continues to increase. Breast cancer is one of the most common causes of mortality in women from all over the world [1]. The disease is characterized by a high heterogeneity, both in molecular terms and in terms of the clinical course and prognosis. In 12%–17% of breast cancer patients, so-called triple-negative breast cancer (TNBC) is diagnosed. One of the treatment methods is chemotherapy. However, chemotherapeutics induce some toxic side effects against normal cells; therefore, they should be used “carefully”. The aim of the recent few years of studies was to develop a therapy that, apart from chemotherapeutics, would also apply the materials of a natural source (e.g., flavonoids), which could increase the quality of the treatment: on the one hand, by a decrease in the proliferation of neoplastic cells and, on the other hand, by weakening the toxic effects of chemotherapeutics on normal tissues [2].

Alternative medicine is becoming more and more popular in the treatment of cancers worldwide. It is estimated that this market has already been extremely profitable. However, the conflicts between oncologists and their patients have also escalated. The former are skeptical when it comes to alternative therapies due to the lack of study results confirming the efficacy of such products; in their mind, the latter ones—the patients—do their best to recover from cancers and return to normal life regardless of the manner to obtain this aim or the costs. Yet, they sometimes are not aware that such supplements may interact with standard chemotherapeutics [3].

One of the natural products that is becoming more and more popular is propolis. It is a sticky substance that originates from floral resins collected by bees (*Apis mellifera*) from the buds and young sprouts of coniferous trees and other green plants. It is supplemented by bees with wax and small amounts of incretion. It may contain some additives, such as pollen or bee bread. The insects use propolis to seal and disinfect hollows and clefts in a hive and to “mummify” the corpses of pests that have entered the hive [4].

There are various types of propolis depending on the latitude where it was collected. There are European, Brazilian, Chinese and New Zealand varieties of propolis. All of them are rich sources of polyphenol compounds. The concentration of these compounds in propolis conditions its anti-microbial [5,6], anti-inflammatory [7], immunomodulatory [8], regenerative [9,10], hepatoprotective [11], antioxidative [12,13] and has antitumor activity [14,15,16,17].

Among over a dozen aromatic esters, the most frequent one is the caffeic acid phenethyl ester (CAPE). This compound was first synthesized in 1988 [18]. It is characterized by strong biotic properties, including the following: antibacterial [19], antiviral [20], anti-inflammatory [21], antioxidative [22], antiplatelet [23] and antitumor [24,25].

*In vitro* studies reveal the cytotoxic properties of CAPE against the cell lines of colorectal carcinoma [26,27], pulmonary carcinoma [28], malignant melanoma [29], gastric carcinoma [30], pancreatic carcinoma [31], hepatic carcinoma [32], cervical carcinoma [33] cholangiocarcinoma [34], glioma [35] and some other cell lines of breast cancer [36,37].

The best known antitumor activity mechanism of the caffeic acid phenethyl ester is its inhibitory activity against the most significant nuclear transcription factor NF-κB. The ability of NF-κB to inhibit apoptosis, proliferation induction and intensification of angiogenesis show that NF-κB may be an important factor in the process of oncogenesis and progression of a cancer. Inhibition of this factor leads to stimulation of apoptosis by an increase of caspase-3 concentration, a decrease of the antiapoptotic protein Bcl-2 and an increase of the proapoptotic protein Bax. All of these changes contribute to an inhibition of the proliferation of the neoplastic cells, as well as tumor regression [38].

The available research data focus mainly on the separate biological effects of propolis of different origin and its selected derivates—caffeic acid, artepillin C, galangin, CAPE and other flavonols or flavonoids—towards malignant cells, rarely evaluating the comparison together of propolis and some composed bioactive compounds. Taking into account the fact that there is lacking research on the anticancer effect of either propolis or CAPE, we have made an attempt to determine *in vitro* whether ethanol extract of propolis and CAPE and may affect the viability and proliferation of triple-negative (estrogen, progesterone and Her-2) MDA-MB-231 and Hs578T human breast cancer cell lines, *versus* the non-cancerous IMR-90 fibroblast line as a control. We provided the concentration/time profiles over selected intervals of time of 24, 48 and 72 h. The results were used for a quantitative assessment of breast carcinoma cells’ viability using the reference MTT and lactate dehydrogenase (LDH) assays. Additionally, the morphology of MDA-MB-231 and Hs578T carcinoma cells was microscopically evaluated with the implementation of the standard hematoxylin and eosin staining protocol.

## 2. Results and Discussion

In recent years, scientists worldwide have been conducting research to find a detailed chemical composition of and the anti-proliferating, cytotoxic and proapoptotic properties of propolis, which is confirmed by the results of various experiments and publications in scientific journals. The resistance of neoplastic cells to standard chemotherapy inspires a continuous search for new compounds with cytostatic activity. One assumption of the chemoprevention concept is to prevent the initiation of cancerogenesis or the inhibition of this process at its early stages. This is aimed at exclusion of the development of a tumor capable of invading neighboring tissues and metastasis. Among the chemopreventive substances, there are non-steroid anti-inflammatory medicines, folic acid, vitamins C and A, vitamin E, carotene, cellulose and many more medicines of a natural origin, including propolis and its components, such as the caffeic acid phenethyl ester.

### 2.1. The Chemical Characterization of Ethanol Extract of Propolis

The identification of chromatographic peaks was accomplished by the information obtained from HPLC-DAD analysis. Reference standards were used for p-coumaric acid, benzoic acid, ferulic acid, gallic acid, caffeic acid, cinnamic acid, apigenin, pinobanksin, kaempferol, kaempferide, acacetin, pinocembrin, galangin, chrysin, quercetin and caffeic acid phenethyl ester. The identification was confirmed by direct comparison of the retention times and spectra acquired in the same analytical conditions. The content of phenolic acids and flavonoid compounds of an ethanolic propolis sample is reported in Table 1. In general, phenolic acids and their esters were the predominant class of substances in ethanol extract of propolis (EEP), followed by flavones and flavonols. Qualitative and quantitative analysis of selected flavonoids and phenolic acids identified pinocembrin, kaempferol, galangin, chrysin, apigenin, quercetin, acacetin, gallic acid, ferulic acid, caffeic acid, caffeic acid phenethyl ester (CAPE), *p*-coumaric acid and cinnamic acid. The flavonoid compounds identified in this study included flavones, flavanones, flavanols, flavonols and chalcones. It is demonstrated that the chemical content of ethanolic extracts of propolis is highly various. The main detected ingredients belonging to phenolic compounds and flavonoids are cinnamic acid and quercetin, respectively. The increasing sequence of quantitative EEP content for selected constituents is presented as follow: flavones < flavonols < aromatic carboxylic acid < phenolic acid.

The complex composition of propolis can be responsible for its multi-directional antiproliferative activity; however, a mixture of different polyphenols in EEP may exhibit the attenuating action between selected constituents, with the partial reduction of their biological activity.

**Table 1:** The chemical characterization and content of the propolis sample. Data gathered by HPLC-DAD analysis. nd, not detected. EEP, ethanol extract of propolis.

Identified Constituents in EEP	Chemical Structure	Retention Time R_t_	Quantity of Crude Propolis (µg∙g^−1^)	Bioactivity
Cinnamic acid	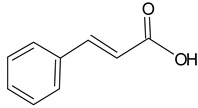	5.20	2432.4	Antimicrobial [39]
p-Coumaric acid	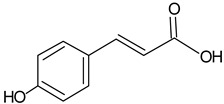	5.69	723.1	Anti-oxidative [40]
Ferulic acid	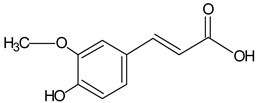	6.23	1559.2	Antimicrobial [41]
Gallic acid	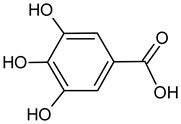	6.98	2041.6	Antimicrobial [42]
Caffeic acid	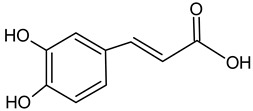	7.36	2317.3	Bacteriostatic [42], anti-inflammatory [43]
Caffeic acid phenethyl ester (CAPE)	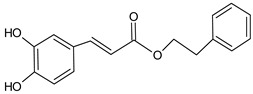	8.63	1356.2	Anti-inflammatory [21], antiviral [20], anticancer [24,25]
Pinobanksin	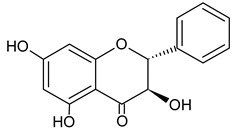	8.97	nd	Antifungal [44]
Kaempferol	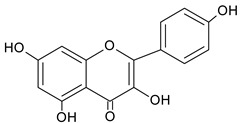	9.34	1874.6	Anti-inflammatory [45]
Apigenin	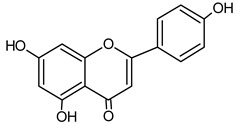	10.40	166.7	Anti-allergic [46], anti-inflammatory [47]
Pinocembrin	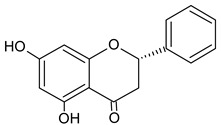	10.92	1557.2	Antifungal [48]
Quercetin	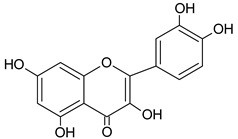	13.19	2047.9	Antioxidative [49]
Chrysin	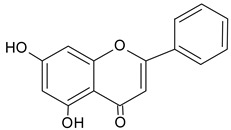	13.93	1147.3	Anti-inflammatory, anticancer [50]
Galangin	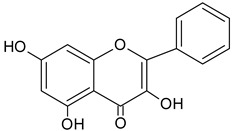	14.72	863.1	Antioxidative [51], antimicrobial [52]
Acacetin	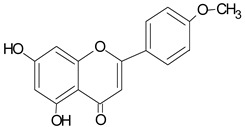	17.96	1007.2	anti-inflammatory [53]
Kaempferide	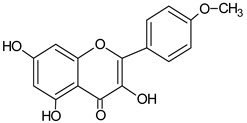	21.67	nd	Antioxidative [54]

### 2.2. The Biological Effects of CAPE and Propolis on Morphological Changes of Hs578T and MDA-MB-231 Breast Carcinoma Cells

The results of our *in vitro* investigation demonstrated that triple-negative MDA-MB-231 and Hs578T human breast carcinoma cells exposed to CAPE and EEP phytochemicals reveal diminished metabolic activity and viability in a dose-dependent and time-dependent manner. Microscopic assessment demonstrated numerous changes in cellular morphology of examined breast carcinoma cells, including a decreased number of affected cells, cell shrinkage and cytoplasmic condensation. These data support the hypothesis that the exposure to some phytochemicals, components of propolis, including derivatives of caffeic acid, may hypothetically reduce the growth of breast cancer cells, compared to the non-cancerous IMR-90 fibroblast control line.

**Figure 1:**
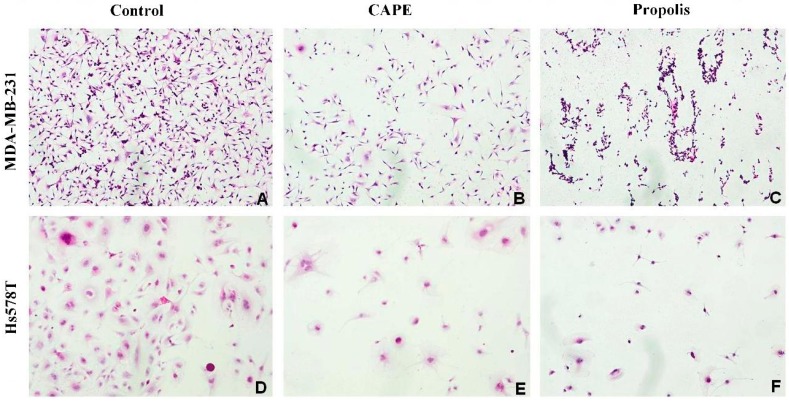
Morphological and cytological features of triple-negative breast cancer (TNBC) MDA-MB-231 (**A–C**) and Hs578T (**D–F**) treated with 0.2% DMSO solution (vehicle control) (A,D), treated with CAPE in a concentration of 80 μM (B,E) and treated with propolis in a concentration of 200.0 μg∙mL^−1^ (C,F) after 72 h of cell exposure to the investigated phytochemicals. (A) MDA-MB-231 grown as a control with DMSO solvent, showing the characteristic monolayer and suspension of carcinoma cells, with the standard features of cellular atypia: nuclear and cytoplasmic pleomorphism, increased nucleus:cytoplasm ratio, highly irregularly-shaped cells (tadpole, caudate), irregular nuclear shapes and hyperchromasia. (B) MDA-MB-231 with CAPE addition shows a decreased number of breast carcinoma cells and necrotic cells in suspension. (C) MDA-MB-231 with propolis addition, showing a decreased number of breast carcinoma cells, cytoplasmic shrinkage, condensed chromatin and stained fragments of decomposed cancer cells. (D) Hs578T in the DMSO solvent, showing blurred cytoplasmic structure and the characteristic future for cellular atypia. (E) Hs578T with CAPE addition, showing a decreased number of cells without significant nucleus and cytoplasmic changes. (F) Hs578T with propolis addition, showing similar, but slightly less obvious morphological changes as for the MDA-MB-231 line, *i.e.*, a decreased number and condensed chromatin.

The 72-h incubation of MDA-MB-231 and Hs578T cells with biologically-active substances—propolis and CAPE—resulted in a decreased number of vital cells, which is shown in Figure 1. Moreover, the cells’ detachment and the changes of their shapes were also observed.

### 2.3. The Assessment of Viability of MDA-MB-231 and Hs578T Cells Exposed to CAPE and EEP with the MTT Assay

**Figure 2:**
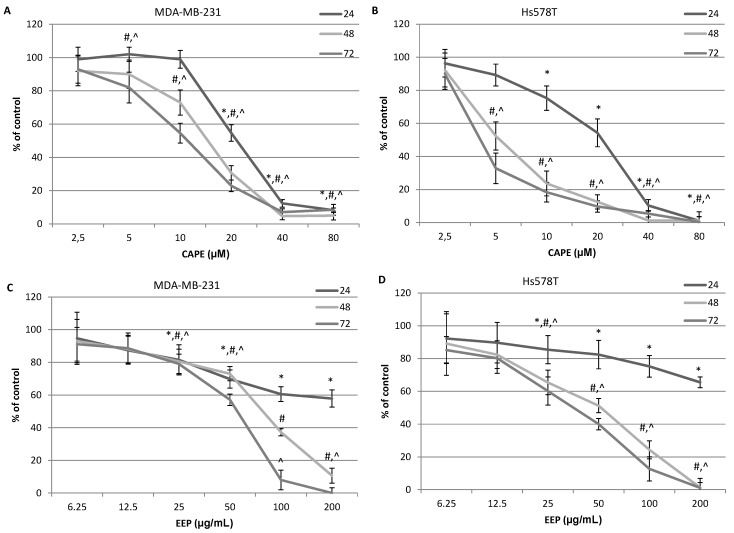
Cytotoxic effects of EEP and CAPE on MDA-MB-231 and Hs578T breast cancer cells. Cells were incubated with 6.25–200.0 μg∙mL^−1^ EEP or with 2.5–80.0 μM CAPE for 24, 48 and 72 h. The values represent the mean ± SD of three independent experiments performed in quadruplicate (*n* = 12). (**A**) Cytotoxic activity of CAPE against MDA-MB-231 cells. (**B**) Cytotoxic activity of CAPE against Hs578T cells. (**C**) Cytotoxic activity of EEP against MDA-MB-231 cells. (**D**) Cytotoxic activity of EEP against Hs578T cells. The percentage of cell death was measured using the MTT cytotoxicity assay. Results are presented as the means of cytotoxicity ± SD. *, ^^^, ^#^ indicate statistically-significant differences compared to the control: * after 24 h of incubation, ^#^ after 48 h and ^^^ after 72 h.

**Figure 3:**
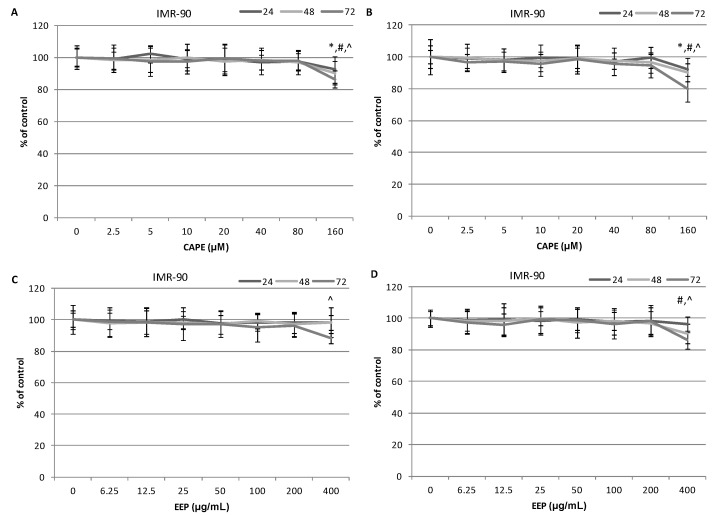
Cytotoxic effects of EEP and CAPE on the normal lung fibroblast IMR-90 cell line. Cells were incubated with 6.25–200.0 μg∙mL^−1^ EEP or with 2.5–80.0 μM CAPE for 24, 48 and 72 h. The values represent the mean ± SD of three independent experiments performed in quadruplicate (*n* = 12). (**A**) Cytotoxic activity of CAPE against IMR-90 cells. The percentage of cell death was measured using the MTT cytotoxicity assay. (**B**) Cytotoxic activity of CAPE against IMR-90 cells. The percentage of cell death was measured using the lactate dehydrogenase (LDH) cytotoxicity assay. (**C**) Cytotoxic activity of EEP against IMR-90 cells. The percentage of cell death was measured using the MTT cytotoxicity assay. (**D**) Cytotoxic activity of EEP against IMR-90 cells. The percentage of cell death was measured using the LDH cytotoxicity assay. Results are presented as the means of cytotoxicity ± SD. *, ^^^, ^#^ indicate statistically-significant differences compared to the control: * after 24 h of incubation, ^#^ after 48 h and ^^^ after 72 h.

The cytotoxic activity of various concentrations of propolis and the caffeic acid phenethyl ester on the breast cancer cells is presented in Figure 2A–D. The viability of MDA-MB-231 and Hs578T cells decreased depending on the concentration and time of exposure to the studied compounds. The cell viability decrease upon exposure to CAPE was stronger for the breast cancer cells of the Hs578T line in the 48th and 72nd hour. In the case of the application of the ethanol extract of the Polish propolis, it could be observed that the cytotoxic activity was stronger against the Hs578T cells than MDA-MB-231 cells, which is presented in Figure 2C,D. The control assay performed with normal fibroblasts of the IMR-90 line showed a slight effect of the studied compounds on the viability and proliferation of the cells in the analyzed concentrations; however, this effect was not statistically significant. Results are presented in Figure 3.

Our results based on MTT and LDH assays are coherent with the data obtained by Wu *et al*. [23] which confirm the hypothesis that breast cancer cells’ viability gradually decreases depending on the increasing dose of CAPE. The estimated IC_50_ value amounted to 15 μM for MDA-MB-231 and MCF-7 cell lines by Wu *et al*. was only slightly higher than the results obtained in our experiment, with 14.08 μM and 8.01 μM for MDA-MB-231 and Hs578T, respectively. The *in vivo* study confirms that administration of CAPE for 3–4 weeks decreases the volume of the tumor in mice from 40%–60% in the case of a heterograft of breast cancer cells of the lines MDA-MB-231 and MCF-7. Interestingly, this study showed that a more aggressive phenotype of breast cancer, MDA-MB-231, was more sensitive to CAPE than the MCF-7 cells. Moreover, both models confirmed that low CAPE doses (approximately 10 nmol CAPE/mouse/day) produce better results for inhibiting the volume of the tumor than much higher doses. The same study also showed that CAPE induces the inhibition of the cell cycle in the S-phase and a complete elimination of breast cancer cells in the G_2_-/M-phase.

### 2.4. The Assessment of the Cytotoxic Activity of CAPE and EEP against MDA-MB-231 and Hs578T Cells with the LDH Assay.

**Figure 4:**
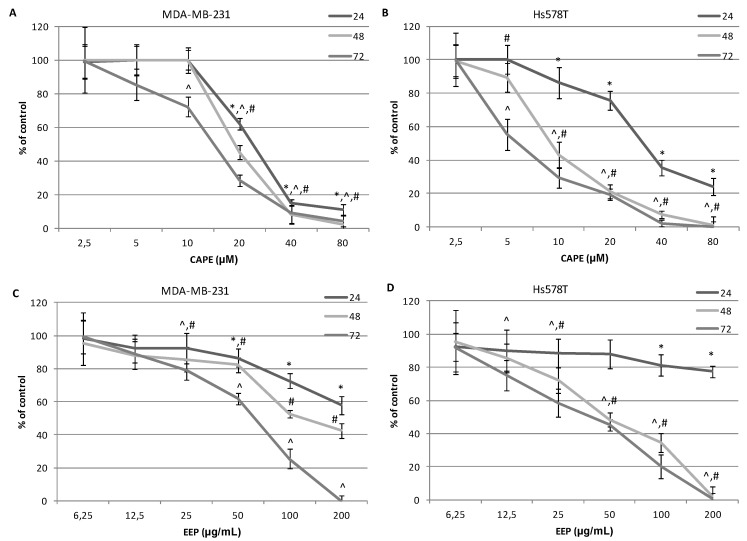
Effect of CAPE and EEP on the viability of MDA-MB-231 and Hs578T cells. The cytotoxicity was evaluated by the LDH assay after 24, 48 and 72 h of incubation of cells with 6.25–200.0 μg∙mL^−1^ EEP or with 2.5–80.0 μM CAPE. The values represent the mean ± SD of three independent experiments (*n* = 12). (**A**) Cytotoxic activity of CAPE against MDA-MB-231 cells. (**B**) Cytotoxic activity of CAPE against Hs578T cells. (**C**) Cytotoxic activity of EEP against MDA-MB-231 cells. (**D**) Cytotoxic activity of EEP against Hs578T cells. The percentage of cell death was measured using the LDH cytotoxicity assay. Results are presented as the means of cytotoxicity ± SD. *, ^^^, ^#^ indicate statistically-significant differences compared to the control: * after 24 h incubation, ^#^ after 48 h and ^^^ after 72 h.

In order to confirm the cytotoxic activity of the studied substances, the method based on enzymatic reactions was applied. This assay assesses the activity of lactate dehydrogenase (LDH), which is a cytosolic enzyme that in physiologic conditions is not released to the environment. The obtained results confirmed that the studied compounds show cytotoxic activity, however not as strongly as could have been concluded from the analysis of the results obtained with the MTT assay. The cytotoxic activity results of the ethanol extract of propolis and the caffeic acid phenethyl ester are presented in Figure 4.

As a result of the conducted experiment assessing the cytotoxic activity of the ethanol extract of propolis and the caffeic acid phenethyl ester towards the triple-negative breast cancer cells, the IC_50_ values were determined for the EEP and CAPE compounds depending on the time of cell exposure (Table 1). The IC_50_ values estimated by the MTT cytotoxicity assay are generally lower than with the lactate dehydrogenase release test. However, the MTT assay determines the mitochondrial activity of cells, which may be interrupted due to the activity of certain chemical compounds on the cells, whereas the LDH assay clearly confirms cell death and is a more precise marker of cell necrosis. It can be observed (Table 2) that the IC_50_ estimations in both assays (MTT and LDH) are higher for MDA-MB-231 cells exposed to CAPE, and a higher sensitivity to the studied compounds was revealed by the breast cancer cells of the Hs578T line (lower IC_50_ values). The IC_50_ after 72 h is almost twice as high for the MDA-MB-231 cells exposed to CAPE than for Hs578T breast cancer cells.

**Table 2:** Comparison of IC_50_ values for MDA-MB-231 and Hs578T cells obtained from the LDH leakage assay and MTT assay following exposure to CAPE and EEP for 24, 48 and 72 h.

Breast Cancer Cell Line (TNBC)	CAPE Exposure, EEP Exposure (h)	MTT Assay IC_50_: CAPE μM (μg∙mL^−1^)	MTT Assay IC_50_: EEP μg∙mL^−1^	LDH Assay IC_50_: CAPE μM (μg∙mL^−1^)	LDH Assay IC_50_: EEP (μg∙mL^−1^)
MDA-MB-231	24	21.05 (5.99)	232.31	22.93 (6.52)	731.68
	48	13.78 (3.92)	63.38	18.64 (5.30)	170.97
	72	11.69 (3.32)	40.40	14.08 (4.00)	48.35
Hs578T	24	16.38 (4.66)	2538.51	32.80 (9.33)	>3000.00
	48	6.60 (1.88)	38.64	11.53 (3.28)	45.07
	72	4.82 (1.37)	31.03	8.01 (2.28)	33.68

The *in vitro* study performed by Omene *et al.* [3] with the use of selected breast cancer cells confirms the substantial cytotoxic effect of CAPE towards the MDA-MB-231, MCF-7 and SK-BR-3 cell lines. The IC_50_ values ranged from 15 μM for MCF-7 up to 35 μM for the MDA-MB-231 line. These data are double those gathered in our study (14.08 μM, MDA-MB-231), which can be related to a different technique used for the cytotoxicity assessment of the investigated phytochemicals. Omene *et al.* also showed that CAPE causes accumulation of the acetylated histone protein by which they suggest that it reveals the properties inhibiting the inhibitor of deacetylation of histones. This mechanism may be partly responsible for the antitumor activity of the caffeic acid phenethyl ester and strengthen its role as a potential antitumor drug. It was also shown that in the lines of breast cancer with a positive expression of the estrogen receptor (ER+), the application of CAPE itself or CAPE found in propolis leads to the decrease in the estrogen and progesterone receptors’ number, which suggests that the decrease of these genes’ expression causes the anti-hormonal effect. Moreover, this study shows that the application of CAPE in the triple-negative breast cancer is possible accompanying hormonal therapy. It was also shown that CAPE inhibits the expression of the mdr-1 gene responsible for the resistance of neoplastic cells to the applied chemotherapeutics.

A similar situation to the caffeic acid phenethyl ester can be observed in the case of the ethanol extract of propolis, which is presented in Figure 4 and Table 2. In this case, the IC_50_ values were also higher for MDA-MB-231 cells; however, these discrepancies were considerably smaller than for CAPE. The exceptions were only the values of IC_50_ obtained in the 24-h incubation of cells with EEP and CAPE. Values for IC_50_ obtained in the LDH test were lower for MDA-MB-231 cells than Hs578T. These finding and the analysis of the IC_50_ values for the investigated substances may also suggest that caffeic acid derivative CAPE has a higher biological activity towards breast carcinoma cells compared to ethanol extract of propolis with a complex chemical composition. Possibly, the “attenuation phenomenon” can be responsible for a less effective action of propolis as a mixture of variable ingredients.

The quantitative composition of propolis is heterogeneous; however, it always contains chemical compounds, such as polyphenols, terpenoids, steroids and amino acids. Propolis samples obtained from various plants may therefore differ in terms of chemical composition. The Polish propolis is mainly classified as the poplar-type propolis, and its dominating components are flavonoids and phenolic compounds, which constitute between 35% and 50%. 

There is more and more proof that the polyphenol compounds found, e.g., in propolis may serve as a supplement to standard chemotherapy and radiotherapy.

Apoptosis is one of the most potent defenses against cancer, because this process eliminates potentially deleterious, mutated cells. Many dietary cancer-preventive compounds, including propolis and its active derivatives, induce apoptosis in cancer cells. The mechanism of evoking apoptosis by propolis seems to be independent of the type of the studied neoplastic cell; however, it is directly dependent on the concentration of the applied extract for the purpose of the study. Some studies suggest that propolis induces apoptosis by releasing cytochrome c from mitochondria to cytosol by means of the caspases’ cascade and with the pro-apoptotic proteins. 

The studies of Watabe *et al.* [36] depict the mechanism of CAPE activity as dependent on the nuclear transcription factor NF-κB. These factors play a significant role in the regulation of death and survival of cells. It is known that the caffeic acid phenethyl ester strongly inhibits NF-κB activation. The study also confirms that CAPE is responsible for the activation of apoptosis in neoplastic cells of various types, but does not lead to the activation of this pathway in the case of the normal fibroblast cells WI-38. It may be caused by the fact that the neoplastic cells with a high base of NF-κB activity are more sensitive to CAPE than normal cells. The study also analyzes the pathways of apoptosis activation in the neoplastic cells. It confirms the activation of apoptosis both in the receptor pathway dependent on the Fas receptor and the mitochondrial pathway with the release of cytochrome c. Additionally, this mechanism may be responsible for the difference in the results regarding the assessment of the cytotoxic activity in the MTT and LDH assays.

Therefore, the results of the experiment conducted by Lee *et al.* [54] are of special interest, since they defined the effect of the caffeic acid phenethyl ester on the activity of proteins p53 and p38 in the C6 glioma cells. This study confirmed that propolis has cytotoxic activity. The scientists proved that CAPE leads to the release of cytochrome c from mitochondrion to cytosol and the activation of caspase-3. What is most important, the expression of the p53 protein, Bax and Bak increased only after 3 h of incubation with CAPE, simultaneously causing a decrease of the expression of the anti-apoptotic protein Bcl-2 upon a 36-h incubation. Similar results were also obtained by Jin *et al.* [55], who carried out an experiment on the line of human myeloid leukemia U937. They proved, similarly to Lee *et al.*, that the caffeic acid phenethyl ester (propolis component) has cytotoxic properties dependent on the concentration and exposure time of the substance on the neoplastic cells. With DAPI staining, they observed in the fluorescent microscope some changes characteristic for apoptosis in the cellular nuclei. They did not confirm the expression of the Fas protein on the surface of the studied cells; however, they observed the release of cytochrome c to cytosol, inhibition of the expression of the anti-apoptotic protein Bcl-2 and an increase of the pro-apoptotic protein Bax. Our results confirm the observations of Jin *et al.*, since, similarly to the Korean team, we revealed that the caffeic acid phenethyl ester and the ethanol extract of the Polish propolis show cytotoxic activity dependent on the concentration of the studied compound and the time of cell exposure to these compounds.

Szliszka *et al*. [56] in their experiment showed that artepillin C found in the Brazilian propolis causes an increase in neoplastic cells’ sensitivity to the TRAIL protein. This protein is a strong stimulator of apoptosis in neoplastic cells and an important factor responsible for the elimination of developing tumors. However, a number of cells undergoing oncogenesis is resistant to the apoptotic death with the participation of the TRAIL protein. Therefore, Szliszka *et al*. decided to sensitize the neoplastic cells to the TRAIL protein by adding substances of a natural origin, such as flavonoids and phenolic acids. In order to confirm the advanced thesis, they used the cytotoxic assays (MTT, LDH), fluorescent staining (to define apoptosis), flow cytometry (to assess the death receptors) and the immune-enzymatic tests. It was proven that the application of the propolis component with the TRAIL protein led to an increase of the number of dying cells though apoptosis by an increase of caspase-3 and -8 activity and inhibition of the nuclear factor NF-κB.

It should be noted that the obtained differences in the MTT and LDH assays suggest the changes at the level of mitochondrial metabolism in the studied compounds. The possible mechanism of the anticancer property of propolis and one of its active derivate, CAPE, seems to be the augmentation of apoptosis phenomenon in human breast cancer cells. The detected changes of carcinoma cells’ morphology following EEP exposure may suggest and indicate the carcinoma cells’ death due to the apoptosis pathway. Therefore, the authors’ next research aim shall be the determination of a hypothetical way for the breast cancer cells’ metabolic death, after exposure to these highly-active phytochemicals inhibiting malignant cells’ metabolism.

## 3. Experimental Section 

### 3.1. Compounds and Reagents

CAPE, MTT, trypan blue, DMEM medium, Leibovitz’s medium, fetal bovine serum (FBS) and tryptic soy broth were purchased from Sigma-Aldrich (St. Louis, MO, USA). Antibiotic/antimycotic, trypsin and phosphate-buffered saline (PBS) were purchased from GE healthcare company (Waukesha, WI, USA). Mayer’s hematoxylin, eosin, xylene and ethanol were obtained from Avantor Performance Materials (Gliwice, Poland).

### 3.2. Propolis Sample Collection and Ethanol Extract of Propolis Preparation

Propolis obtained from the bee *Apis mellifera* was collected in Kamianna in the south of Poland in the Małopolskie Province. It was stored at 4 °C out of the light until the extraction. In order to obtain the ethanol extract, 10 g of raw propolis were minced, and 100 g of 75% ethanol were added. The tightly closed flask was placed in a shaker for two weeks at room temperature, out of the light. After this time, the extract was cooled at 4 °C for 24 h in order to remove all of the substances that are insoluble in ethanol. Next, it was filtered under lower pressure. The obtained filtrate was vaporized with a vacuum evaporator at 40 °C. In the next stage, the obtained extract was placed in an incubator for 7 days in order to vaporize the ethanol residues. The content of the flask with the known extract mass was diluted in a pure dimethyl sulfoxide until the working concentration of 100 mg∙mL^−1^ was obtained.

### 3.3. High-Performance Liquid Chromatography with Diode Array Detector Analysis

To determine the chemical composition of EEP, a high performance liquid chromatography method was applied with the use of a Varian 920-LC HPLC (Harbor City, CA, USA), equipped with a 900-LC model autosampler, gradient pump, 330 model DAD and the Galaxie software (Version 1.9 SP3, Agilent Technologies, Santa Clara, CA, USA) for data acquisition and processing. The analysis was carried out according to a protocol described in the study [5]. Reference substances in ethanol solution (0.1 mg∙mL^−1^) containing acacetin, apigenin, chrysin, galangin, kaempferide, kaempferol, pinobanksin, pinocembrin, quercetin, caffeic acid, cinnamic acid, *o*-coumaric acid, *m*-coumaric acid, *p*-coumaric acid, gallic acid, ferulic acid and caffeic acid phenylethyl ester (CAPE) were prepared from standard stock solutions. Phenolic compounds were purchased from Carl Roth GmbH (Karlsruhe, Germany) and Sigma Chemical Company (St. Louis, MO, USA).

### 3.4. Triple-Negative Breast Cancer Cell Cultures

Cell lines of a human breast (mammary gland) cancer were cultured in accordance with the manufacturer’s recommendations. The metastatic human breast cancer line MDA-MB-231 (adenocarcinoma) derived from metastatic site pleural effusion (Catalogue No. ATCC HTB-26, American Type Culture Collection, Manassas, VA, USA) was cultured with Leibovitz’s l-15 medium with 10% of inactivated fetal bovine serum (FBS, Sigma-Aldrich) at 37 °C without CO_2_. Breast cancer cells Hs578T (Catalogue No. ATCC HTB-126 American Type Culture Collection, Manassas, VA, USA) were cultured on Dulbecco’s modified Eagle’s medium with 10% inactivated fetal bovine serum with bovine insulin at a final concentration of 0.01 mg∙mL^−1^. The cells were incubated in a CO_2_ incubator at 37 °C and in the air atmosphere containing 5% CO_2_. The studied cell lines were supplemented with antibiotics with the following final concentrations: penicillin 100 U∙mL^−1^, streptomycin 100 µg∙mL^−1^ and a fungistatic amphotericin B with a concentration of 0.25 µg∙mL^−1^. The medium was changed every 2–3 days, and the passage was carried out with a confluence of 80%–90%.

### 3.5. Microscopic Evaluation of Carcinoma Cells Morphology 

The cells of the studied lines were inoculated onto 2-chamber microscopic culture vessels (Lab-Tek^TM^, Waltham, MA, USA) in the amount of 1000 cells per a well and were left for 24 h in order to obtain the logarithmic growth. After this time, the studied compounds were added to the media in appropriate concentrations and were left for 24, 48 and 72 h, respectively, depending on the time of the experiment. After a defined time period, they were fixed for 12 h in 96% ethanol. Then, the cells were hydrated in the following series of dilutions: 99.6%, 96%, 90%, 80%, 70% and 50% and stained with hematoxylin for 12 min (standard H&E staining protocol). Next, the plates were washed with the PBS solution for approximately 30 min in order to blue up and then were incubated for 30 s with eosin. The plates were washed with PBS solution again and dehydrated with ethanol of increasing concentrations of 50%, 70%, 80%, 90%, 96% and 99.6%. In the next stage, the plates were immersed in the ethanol and xylene mixture (50:50) for 1 min and then in pure xylene. The plates were mounted and analyzed under a microscope.

### 3.6. The Initial Evaluation of Viability of Triple-Negative Breast Cancer Cells

The trypan blue viability assay was applied in order to determine the efficient and optimal ranges of concentrations of CAPE and EEP. The cells were inoculated on the 96-well plates with a density of 5 × 10^3^ per 100 µL of medium and were left to stick for 12 h. Then, the cells were treated with the solvent or the active substances for 24 h and later with the use of maximum ranges of concentrations from 0–320 µM and 0–400 µg∙mL^−1^ for CAPE and propolis, respectively. The tested and chosen concentrations for CAPE were 2.5, 5, 10, 20, 40 and 80 µM and for propolis were: 6.25, 12.5, 25, 50, 100 and 200 µg∙mL^−1^. Investigated cells were trypsinized, centrifuged and stained with the trypan blue solution 0.04% (w/v, Sigma-Aldrich) and counted with the automatic cell counter (TC20^TM^ Automated Cell Counter Bio-Rad, Hercules, CA, USA), in accordance with the manufacturer’s protocol. Cell viability was presented as a percent mean ± SEM with the three-times independently repeated experiments from three internal measurements in each.

### 3.7. MTT Cell Proliferation and *Cytotoxicity Assay*

The MTT assay is a colorimetric method, which enables assessing the cell metabolic activity. It consists of a colorimetric determination of a colored product, which originates after bromide is added (3(4,5-dimethyltiazol-2-yl)-2,5-diphenyl tetrazolium (MTT) to the cell culture in the presence of the tested substances. The amount of the originating formazan is proportional to the amount of living cells.

In order to determine the cytotoxicity of the studied compound, the cells were inoculated on the 96-well plates in the amount of 10^4^ cell/well, and fresh medium was added and left for 72 h to obtain the logarithmic cell growth. At this time, the medium was decanted, and a culture medium containing EEP and CAPE was added in the analyzed concentrations prepared in the course of a series of dilutions in the culture medium. Zero-point-one milliliters of medium with a defined concentration of the studied substance were added to each well and left for 24 h in the CO_2_ incubator at 37 °C in a steam atmosphere with 5% of CO_2_ or in the thermostat in the case of the MDA-MB-231 cells.

At this time, the medium was decanted, and 100 μL of 1% MTT solution in the culture media were added. The cells were incubated with a reagent for 4 h in the incubator at 37 °C. At this time, the MTT solution was decanted from the crystals, and the obtained formazan crystals were diluted by adding to each well 200 μL of DMSO. The whole was shaken evenly to dilute the obtained crystals. To read the absorbance, the ELISA plate reader by BioTek was used with a wavelength of 570 nm.

The solvent control was the absorbance readings obtained from the plates where the cell lines were cultured without the studied substances containing the additionally-applied solvents (dimethyl sulfoxide with a concentration of 0.2%). This solvent control protocol was applied to both the MTT and the LDH assay.

### 3.8. Lactate Dehydrogenase Release Assay

Upon reaching the almost confluent state, the cells were washed twice with the DPBS and removed from the medium with 0.25% solution of trypsin in EDTA. The detached cells were centrifuged at 1000× *g* for 3 min at room temperature (25 °C). The obtained cell sediment was suspended in the appropriate amount of assay medium with 1% of the fetal bovine serum in such a way that the cell concentration was 1 × 10^5^ cells/ml. One hundred microliters of cell suspension were added to each well of the medium plate (96 wells), except the wells containing control I (background) and 100 μL of the assay medium. To assess the degree of cytotoxicity, three types of controls were performed:

(i) Background control (Control I): 200 μL assay medium per well;
(ii) Low control (Control II = spontaneous LDH release): 100 μL assay medium and 100 μL cell suspension;
(iii) High control (Control III = maximum LDH release): 100 μL Triton-X solution (final concentration 2%) in assay medium and 100 μL cell suspension per well.

Additionally, controls of the tested substances were performed: 100 μL of assay medium and 100 μL of the tested substance at the highest possible concentration without cells (separately for propolis and CAPE). Then, the cells were incubated for 48 h in the incubator at 37 °C in a steam atmosphere with 5% CO_2_. Directly prior to use, dilutions were prepared of the tested substances in the assay medium in such a way that the final volume of all of the ingredients in the well was 200 μL. The assay medium was removed from above the cells attached to the bottom of the wells, and 100 μL of fresh assay medium were added to each well, as well as 100 μL of the tested substance in appropriate concentrations. The cells were incubated at 37 °C in a steam atmosphere with 5% CO_2_ depending on the experimental needs for 24 and 48 h. At the defined incubation time, 100 μL of the supernatant were collected from each plate and moved to the micro test plates (96 wells), and 100 μL of reaction mixture were added. The micro test plates were incubated at room temperature for 30 min without light. At the defined time, the absorbance measurement of each sample was performed with the ELISA reader with a wavelength 490 nm. The length of the reference wave was 600 nm. All of the samples were performed in triplicate. The value of background control absorbance must be subtracted from each absorbance value, and the obtained values should be used in the following formula:

Cytotoxicity% = (absorbance value of the studied sample − absorbance value of Control II)/absorbance value of Control III − absorbance value of Control II

### 3.9. Statistical Analysis

The statistical analysis was performed with the Statistica software (Version 8.0, StatSoft Poland). The obtained results were shown in the form of the mean and standard variations. The obtained results were repeated in 4 independent experiments (*n* = 96 for each studied concentration for the MTT assay and *n* = 12 for each studied concentration for the LDH assay). The normality of the distribution was defined with the Shapiro-Wilk test. In order to define the statistical significance, the Student *t*-test was used, as well as the one-parameter ANOVA. Statistical significance was accepted at *p* < 0.05.

## 4. Conclusions 

Based on our findings, propolis and the caffeic acid phenethyl ester substantially inhibit the growth of the cells of triple-negative breast cancer of the lines MDA-MB-231 and Hs578T. The cytotoxic activity of the studied compounds depends on the time of exposure and the concentration of the caffeic acid phenethyl ester and ethanol extract of propolis. A more detailed study should reveal whether CAPE, as a component of propolis, might potentially be applied as a therapeutic medium or as an adjuvant for conventional chemotherapeutics, potentiating their biological, antiproliferative effects.
